# High-Throughput Analysis of Gene Function in the Bacterial Predator Bdellovibrio bacteriovorus

**DOI:** 10.1128/mBio.01040-19

**Published:** 2019-06-11

**Authors:** Miles C. Duncan, Rebecca K. Gillette, Micah A. Maglasang, Elizabeth A. Corn, Albert K. Tai, David W. Lazinski, Robert M. Q. Shanks, Daniel E. Kadouri, Andrew Camilli

**Affiliations:** a Department of Molecular Biology and Microbiology, Tufts University School of Medicine, Boston, Massachusetts, USA; b Department of Integrative Physiology and Pathobiology, Tufts University School of Medicine, Boston, Massachusetts, USA; c Department of Ophthalmology, Campbell Laboratory of Ophthalmic Microbiology, Fox Center for Vision Restoration, University of Pittsburgh, Pittsburgh, Pennsylvania, USA; d Department of Oral Biology, Rutgers School of Dental Medicine, Newark, New Jersey, USA; University of Georgia; The Hebrew University of Jerusalem; Sapienza University of Rome

**Keywords:** *Bdellovibrio bacteriovorus*, *Escherichia coli*, Tn-seq, *Vibrio cholerae*, predatory bacteria

## Abstract

Bdellovibrio bacteriovorus is a bacterial predator capable of killing and replicating inside most Gram-negative bacteria, including antibiotic-resistant pathogens. Despite growing interest in this organism as a potential therapeutic, many of its genes remain uncharacterized. Here, we perform a high-throughput genetic screen with B. bacteriovorus using transposon sequencing (Tn-seq) to explore the genetic requirements of predation. Two hundred one genes were deemed essential for growth in the absence of prey, whereas over 100 genes were found to be specifically required for predative growth on the human pathogens Vibrio cholerae and Escherichia coli in both planktonic and biofilm states. To further this work, we created an ordered-knockout library in B. bacteriovorus and developed new high-throughput techniques to characterize the mutants by their stage of deficiency in the predator life cycle. Using microscopy and flow cytometry, we confirmed 10 mutants defective in prey attachment and eight mutants defective in prey rounding. The majority of these genes are hypothetical and previously uncharacterized. Finally, we propose new nomenclature to group B. bacteriovorus mutants into classes based on their stage of predation defect. These results contribute to our basic understanding of bacterial predation and may be useful for harnessing B. bacteriovorus to kill harmful pathogens in the clinical setting.

## Abstract (precis)

Bdellovibrio bacteriovorus is a predatory bacterium that can kill a wide range of Gram-negative bacteria, including many human pathogens. Given the global rise of antibiotic resistance and dearth of new antibiotics discovered in the past 30 years, this predator has potential as an alternative to traditional antibiotics. For many years, B. bacteriovorus research was hampered by a lack of genetic tools, and the genetic mechanisms of predation have only recently begun to be established. Here, we comprehensively identify and characterize predator genes required for killing bacterial prey, as well as genes that interfere in this process, which may allow us to design better therapeutic predators. Based on our study, we and other researchers may ultimately be able to genetically engineer strains that have improved killing rates, target specific species of prey, or preferentially target prey in the planktonic or biofilm state.

## INTRODUCTION

Bdellovibrio bacteriovorus, first discovered in 1962, is a small predatory Gram-negative bacterium that is ubiquitous in soil and aquatic environments (1). As B. bacteriovorus kills over 100 Gram-negative pathogens, researchers see potential in this predator as a “living antibiotic” (2, 3). In addition to being able to disrupt bacterial biofilms, the organism is poorly immunogenic to mammals, likely due to its sheathed flagellum and unusual lipopolysaccharide composition (4). Two recent studies have made use of these features, deploying the predator to clear Klebsiella pneumoniae and Shigella flexneri infections *in vivo* (5, 6). B. bacteriovorus is also potent against Vibrio cholerae and Escherichia coli, two causative agents of severe diarrheal disease (7). Given the alarming rise of antibiotic-resistant pathogens and paucity of new anti-infectives, B. bacteriovorus is well positioned as an alternative to traditional antibiotics (8, 9).

In the wild, B. bacteriovorus uses chemotaxis and a single polar flagellum to hunt groups of prey bacteria (10–12). Once in close proximity, B. bacteriovorus collides with individual prey and attaches through an unknown mechanism (13, 14). Next, B. bacteriovorus invades the prey periplasm, likely through use of retractable pili, and secretes hydrolytic enzymes that kill the prey within 10 to 20 min of invasion (11, 15). The predator subsequently remodels host peptidoglycan to form the spherical bdelloplast, where it degrades intracellular contents to fuel its own filamentous growth (16). Finally, 3 to 4 h following initial contact, the prey cell is lysed, and four to six daughter cells emerge from their protected niche.

Despite this well-documented life cycle, relatively little is known of the genetic mechanisms underlying predation. Until recently, genetic screens in B. bacteriovorus were intractable, meaning studies of molecular mechanisms have lagged behind those of other model organisms (17, 18). To date, over 40% of its genome consists of uncharacterized or hypothetical genes, likely owing to its unique lifestyle and distant relationship to model bacteria like E. coli.

Here, we report the results of forward genetic screens to identify B. bacteriovorus genes required for predation and the creation of an ordered-knockout library to facilitate further the study of this organism. We also establish several assays for high-throughput characterization of predator gene function and confirm the stage of predation deficiency for 11 B. bacteriovorus mutants. These findings contribute to our basic understanding of this predatory life cycle and may be useful in genetic engineering of B. bacteriovorus strains for use in the clinic.

## RESULTS

### Transposon sequencing for B. bacteriovorus genes involved in predation.

To identify predator genes and pathways playing a role in predation, we first created a transposon library in a host-independent (HI) mutant of B. bacteriovorus 109J (see Data Set S1 in the supplemental material) using the mariner delivery vector pBT20 (19). Unlike the wild-type (WT) bacterium that requires predation for growth, this axenic mutant can also grow in a complex medium (peptone-yeast extract [PYE]) in the absence of prey. HI mutants are known to be slower predators, as they are deregulated in the transition from growth to attack phase (Fig. S1). However, we could not use the WT obligate predator background for our screen, as transposon insertions in predation-essential genes would be nonviable. The B. bacteriovorus library contained 90,000 unique insertions, which is saturating for a genome of 3,584 genes. Following growth on PYE plates, we pooled the colonies and infected 10^10^ CFU of planktonic V. cholerae and E. coli at a multiplicity of infection (MOI) of 0.001 and 10^10^ biofilm prey at an MOI of 0.01. These low MOIs ensured several rounds of replication, allowing greater separation between neutral and low- or high-fitness mutants. We observed 99% planktonic and biofilm prey killing for V. cholerae and E. coli after 48 h and 30 h of infection, respectively.

10.1128/mBio.01040-19.1 FIG S1 Prey rounding by wild-type and host-independent B. bacteriovorus. Prey V. cholerae cells were scored for rounding at 1, 2, or 3 hours postinfection with B. bacteriovorus. Download FIG S1, TIF file, 0.4 MB. Copyright © 2019 Duncan et al. 2019 Duncan et al. This content is distributed under the terms of the Creative Commons Attribution 4.0 International license.

10.1128/mBio.01040-19.4 DATA SET S1 Strains used in this study. Download Data Set S1, XLS file, 0.1 MB. Copyright © 2019 Duncan et al. 2019 Duncan et al. This content is distributed under the terms of the Creative Commons Attribution 4.0 International license.

We next isolated genomic DNA (gDNA) from each sample, which included three biological replicates of B. bacteriovorus inputs, as well as three biological replicates of B. bacteriovorus grown on planktonic and biofilm V. cholerae (VCPL and VCBF, respectively) and planktonic and biofilm E. coli (ECPL and ECBF, respectively). We then used Nextera transposon sequencing (Tn-seq) (20) to process the gDNA for massively parallel sequencing of the mariner transposon junctions. By sequencing using the Illumina HiSeq 2500 platform, we determined the frequency of each transposon insertion in the B. bacteriovorus inputs or prey-outgrown populations. We calculated the fitness contribution of each gene using bioinformatics software, as previously described (21), where fitness represents the net survival of that gene disruption mutant relative to the bulk population (Data Set S2). A total of 201 genes are putative essentials for axenic growth (Data Set S3). Many of these are housekeeping genes involved in fundamental processes like transcription, translation, replication, division, and membrane/cell wall biogenesis and were not studied further.

10.1128/mBio.01040-19.5 DATA SET S2 Fitness values from the full Tn-seq screens. Fitness (*w*) and standard deviations for three biological replicates are shown. Fitness values below 0.5 are highlighted in blue, and those above 2.0 are highlighted in orange. Download Data Set S2, XLS file, 0.9 MB. Copyright © 2019 Duncan et al. 2019 Duncan et al. This content is distributed under the terms of the Creative Commons Attribution 4.0 International license.

10.1128/mBio.01040-19.6 DATA SET S3 List of putative essential genes for HI B. bacteriovorus growth. Genes with zero insertions are listed in rows 2 to 202, and genes averaging less than 1 insertion are shown in rows 203 to 276. Download Data Set S3, XLS file, 0.1 MB. Copyright © 2019 Duncan et al. 2019 Duncan et al. This content is distributed under the terms of the Creative Commons Attribution 4.0 International license.

**FIG 1:**
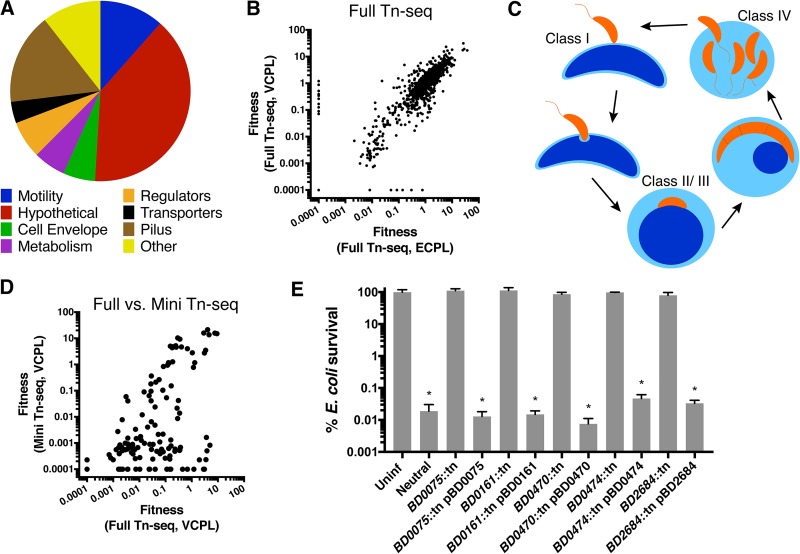
Identification of B. bacteriovorus mutants with altered fitness in bacterial predation. (A) We generated a complex B. bacteriovorus transposon mutant library and subjected it to transposon insertion sequencing (Tn-seq) before and after expansion on V. cholerae and E. coli. Mutants with decreased fitness (*W *<* *0.1) are shown and categorized according to gene ontology terms. (B) A correlation plot of fitness values for B. bacteriovorus preying on planktonic V. cholerae (VCPL) or E. coli (ECPL) in the full Tn-seq. Each dot represents the fitness values for one gene against the two-prey species on the *x* and *y* axes. (C) Diagram of the B. bacteriovorus predatory life cycle. Mutants can show defects in prey attachment (class I), killing (class II), rounding (class III), or exit from prey (class IV). (D) Correlation plots of fitness values comparing results from the full- and mini-Tn-seq screens. (E) E. coli survival following infection with B. bacteriovorus mutants or complemented strains. The average E. coli survival percentage and standard errors of the mean (SEM) for three biological replicates are shown. Significance was determined by comparing E. coli survival against each strain compared to the uninfected (Uninf) control. *, *P < *0.001 (ANOVA and Dunnett’s multiple-comparison test).

Using gene ontology terms, we characterized the 104 insertions that reduced B. bacteriovorus fitness (*W*) to <0.1 when preying on planktonic V. cholerae (Fig. 1A). This list was similar to the genes required for B. bacteriovorus replication under the three other conditions, VCBF, ECPL, and ECBF (Fig. 1B). With 41 genes, hypothetical was the most abundant gene category. Other prominent categories included pilus (type IVa and IVb/FLP), motility, cell envelope, and metabolism genes. Many of these genes were previously characterized as required for predation; flagellar motility is important for efficient encounters with prey (10), and pili are hypothesized to aid in prey entry following attachment (Fig. 1C) (13, 22, 23).

### Creation of B. bacteriovorus ordered-knockout library and hit validation.

To validate B. bacteriovorus hits from the initial Tn-seq, we aimed to create a smaller library of representative mutants and repeat the screen as a mini-Tn-seq. To that end, we created an ordered-knockout library in B. bacteriovorus using previously published techniques (24, 25). We used colony PCR to confirm the identity of mutants of interest for follow up and were able to pool 80 of the top 104 hits from the initial VCPL Tn-seq. We also added 40 borderline hits, with fitness scores ranging from 0.1 to 0.5, 12 mutants with improved fitness (*W *>* *3), and several mutants with defects specific to replication on biofilm prey or E. coli.

We carried out the mini-Tn-seq as described above for the full Tn-seq, repeating the screen with a smaller pool of 141 defective mutants and several intergenic insertion mutants as neutral controls, for VCPL, VCBF, ECPL, and ECBF prey (Data Set S4). For VCPL, the full- and mini-Tn-seq results were modestly correlated, with a rho (*r_s_*) value of 0.37 by Spearman’s correlation (Fig. 1D). When points deviated from the diagonal, they mostly fell below the line and not above it, indicating that defects originally observed in the full Tn-seq were more severe in the mini-Tn-seq. Although the reasons for this are unclear, smaller mutant pools often exacerbate fitness defects; in this case, the mini-Tn-seq had 600-fold fewer mutants than did the full Tn-seq. In a comparison of two mini-Tn-seq conditions, mutant fitness values showed stronger correlations, with VCPL versus VCBF having an *r_s_* of 0.79, VCPL versus ECPL having an *r_s_* of 0.82, and ECPL versus ECBF having an *r_s_* of 0.83. This suggests that while most B. bacteriovorus predation genes are nonspecialized, certain genes may be more important for predation on specific prey or prey states.

10.1128/mBio.01040-19.7 DATA SET S4 Fitness values from the mini-Tn-seq screens. Fitness (*w*) and standard deviation for three biological replicates are shown. Fitness values below 0.5 are highlighted in blue, and those above 2.0 are highlighted in orange. Fitness values for the full-Tn-seq conditions of VCPL and VCBF are shown for reference. Gene annotations, predicted functions, and notes are listed in columns M to P. Download Data Set S4, XLS file, 0.1 MB. Copyright © 2019 Duncan et al. 2019 Duncan et al. This content is distributed under the terms of the Creative Commons Attribution 4.0 International license.

To confirm these fitness defects were due to disruption of the gene of interest, and not off-target effects, we complemented five mutant strains and tested them for prey killing (Fig. 1E). The *BD0075*::tn, *BD0161*::tn, *BD0470*::tn, *BD0474*::tn, and *BD2684*::tn mutant strains were unable to kill E. coli, and prey survival was not significantly changed from that in the uninfected E. coli control (*P > *0.77). However, the plasmid-complemented strains showed 3- to 4-log killing of prey E. coli, which was indistinguishable from the neutral B. bacteriovorus mutant control results (*P = *0.99).

### FACS with Tn-seq to identify genes required for prey attachment and validation.

**FIG 2:**
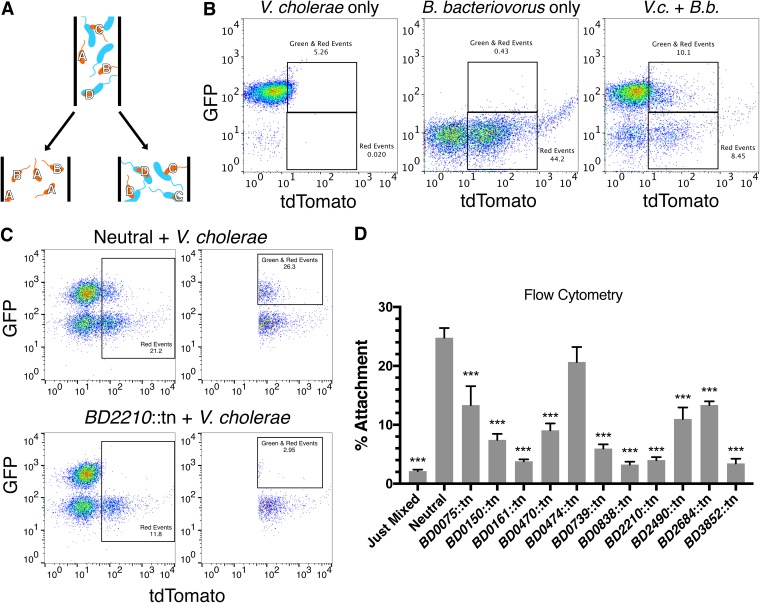
Identification of B. bacteriovorus mutants with attachment defects by Tn-FACSeq. (A) Schematic of the Tn-FACSeq screen. Following a 3-h infection, red fluorescent B. bacteriovorus are sorted into two pools based on whether they associate with green fluorescent V. cholerae or not. In this example, strains A and B do not attach, while strains C and D do. (B) Example gating strategy used to sort B. bacteriovorus by Tn-FACSeq. *V.c.*, V. cholerae; *B.b.*, B. bacteriovorus. (C) Gating strategy for flow cytometry-based validation of attachment-defective mutants identified in Tn-FACSeq. The left panels gate all red events, and the right panels gate all events that are red and green from the same experiment. (D) Quantification of the flow cytometry results in panel C. The average attachment percentage and standard errors of the mean (SEM) for three to four biological replicates are shown. Significance was determined by comparing each strain’s attachment percentage to that of the neutral control. ***, *P < *0.001 (ANOVA and Dunnett’s multiple-comparison test).

Given the large number of validating hits, we prioritized high-throughput characterization of B. bacteriovorus gene function into classes by stage of predation deficiency (Table 1 and Fig. 1C). We hypothesized that mutants could show defects in prey attachment (class I), killing (class II), rounding (class III), or exit of daughter cells from prey (class IV). Previously, we developed a flow cytometry-based assay to measure predator-prey interactions using green fluorescent protein (GFP)-expressing V. cholerae and WT B. bacteriovorus carrying a tdTomato expression vector (20). We reasoned that we could use fluorescence-activated cell sorting (FACS) on our mini B. bacteriovorus transposon mutant library to distinguish mutants that could still attach to prey from those that could not (Fig. 2A).

**TABLE 1:** Proposed classification scheme for B. bacteriovorus mutants

Class	Activity by predation deficiency^*a*^			
	Attach	Kill	Round	Exit
I	**−**	**−**	**−**	**−**
II	**+**	**−**	**−**	**−**
III	**+**	**+**	**−**	**−**
IV	**+**	**+**	**+**	**−**

a **−**, no activity; +, activity.

For this technique, which we termed Tn-FACSeq, we first designed a new TdTomato expression vector and transformed it *en masse* into our B. bacteriovorus mutant pool used previously in the mini-Tn-seq (Fig. 1D). We grew the B. bacteriovorus mutant pool for 4 days on petri plates, at which point we started overnight cultures of the mutant pool and GFP-expressing V. cholerae cells. The next day, we infected V. cholerae with the B. bacteriovorus pool at an MOI of 1 for 3 h at 30°C, with shaking. For each replicate, we sorted the samples for 2 h into a red-only tube (B. bacteriovorus only) or a green and red tube (B. bacteriovorus attached to V. cholerae) (Fig. 2B). We plated the sorted populations and pooled the B. bacteriovorus colonies after 8 days of growth. V. cholerae could not grow on these plates due to the addition of gentamicin and chloramphenicol. We then isolated gDNA and used Tn-seq, as described above, to determine the relative abundance of each mutant in the attachment-positive or attachment-deficient populations (Data Set S5).

10.1128/mBio.01040-19.8 DATA SET S5 Summarized results from Tn-FACSeq, killing assay, and Tn-SphereSeq. Fitness values for the full- and mini-Tn-seq screens on VCPL are shown for reference. For all assays, scores or survival percentages are shown, along with standard deviations and the number of biological replicates (*n*). For Tn-FACSeq, attachment scores less than 0.5 are highlighted in yellow. For survival, E. coli survival rates less than 1% are highlighted in yellow. For Tn-SphereSeq, rounding scores less than 0.4 are highlighted in yellow. Download Data Set S5, XLS file, 0.1 MB. Copyright © 2019 Duncan et al. 2019 Duncan et al. This content is distributed under the terms of the Creative Commons Attribution 4.0 International license.

As in traditional Tn-seq, mutants with attachment scores near 1.0 show no attachment defects. Of the 104 B. bacteriovorus mutants tested, 34 had attachment scores below 0.5, and 16 had attachment scores below 0.3. Intergenic insertion mutants, serving as a control, collectively had an attachment score of 0.97. Mutants with low scores had insertions in genes for type IV pili, adventurous gliding motility, and many hypotheticals.

To validate the Tn-FACSeq hits, we isolated attachment-defective mutants from the ordered library and tested them individually for attachment by flow cytometry (Fig. 2C and D and S2). As a neutral control, we chose the *BD0604*::tn mutant, which has an insertion in a nonfunctional flagellin gene (10). This mutant also showed fitness scores close to 1.0 in the full- and mini-Tn-seq screens. While 25% of fluorescent *BD0604*::tn cells were attached to V. cholerae under these conditions, this number was significantly lower for nearly all mutants tested (*P = *0.0001, Fig. 1D). The exception was the *BD0474*::tn mutant, an uncharacterized FHA domain-containing protein (*P = *0.32).

**FIG 3:**
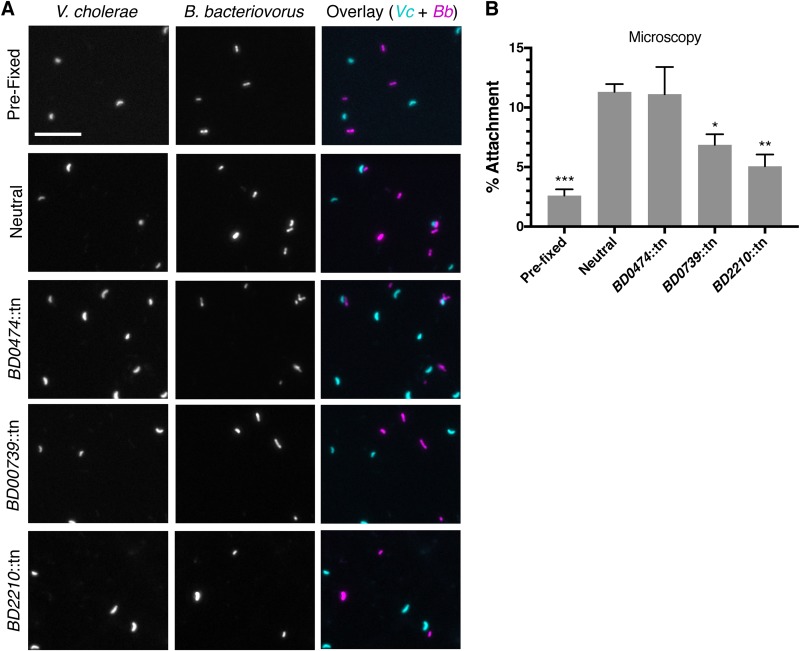
Microscopy validation of Tn-FACSeq results. (A) Fluorescent microscopy images of V. cholerae (cyan) and B. bacteriovorus (magenta) following infection. Scale bar = 10 μm. (B) Quantification of the results in panel A. A minimum of 1,100 B. bacteriovorus cells were scored under each condition, double blind, for attachment to V. cholerae. The average B. bacteriovorus attachment percentage and standard errors of the mean (SEM) for three biological replicates are shown. Significance was determined by comparing each strain’s attachment percentage to that of the neutral control. *, *P < *0.0469; **, *P < *0.0061; ***, *P < *0.0002 (ANOVA and Dunnett’s multiple-comparison test).

To further validate these results, we selected a subset of mutants to test for attachment by microscopy. After imaging the slides, we scored all fluorescent B. bacteriovorus cells in double-blind fashion, for attachment (Fig. 3A and B). As in the flow cytometry experiment, the *BD0739*::tn and *BD2210*::tn mutants demonstrated significantly reduced attachment, while the *BD0474*::tn mutant did not. This supported our flow cytometry results.

### Prey killing assay to identify genes required for B. bacteriovorus to kill E. coli.

Following attachment, B. bacteriovorus invades the prey periplasm and begins to secrete toxic hydrolytic enzymes. Prey death occurs 10 to 20 min following predator entry, just before the prey are remodeled into spherical bdelloplasts (9, 11). We sought to characterize whether the predation-deficient mutants could still kill their prey and thus might have a defect at a later stage of predation, such as intracellular replication or prey exit.

To test individual mutants, we repeated the infection steps used above for the Tn-seq screens with planktonic E. coli at an MOI of 1. After a 30-h infection, we plated for surviving prey and compared the colony counts to uninfected E. coli or E. coli infected with the neutral *BD0604*::tn mutant. After separately testing 47 mutants, we found that 34 did not kill prey (>50% survival), six plus the *BD0604*::tn control strain still killed prey (<1% survival), and another six showed intermediate results (survival between 50% and 1%) (Data Set S5, column I). Of the six B. bacteriovorus mutants that could still kill prey, three were predation defective in the full Tn-seq screen but did not validate in the mini-Tn-seq.

### Tn-SphereSeq to identify genes required for prey rounding and validation.

To continue high-throughput characterization of B. bacteriovorus gene function, we designed another new assay, termed Tn-SphereSeq, to rapidly identify mutants that could no longer round their prey into spherical bdelloplasts. Prey rounding is a critical step in the B. bacteriovorus life cycle (Fig. 1C), and rounding-deficient mutants should be severely attenuated. To identify these mutants, we coupled traditional Tn-seq and a previously described protocol to isolate B. bacteriovorus-containing bdelloplasts by differential centrifugation (26).

**FIG 4:**
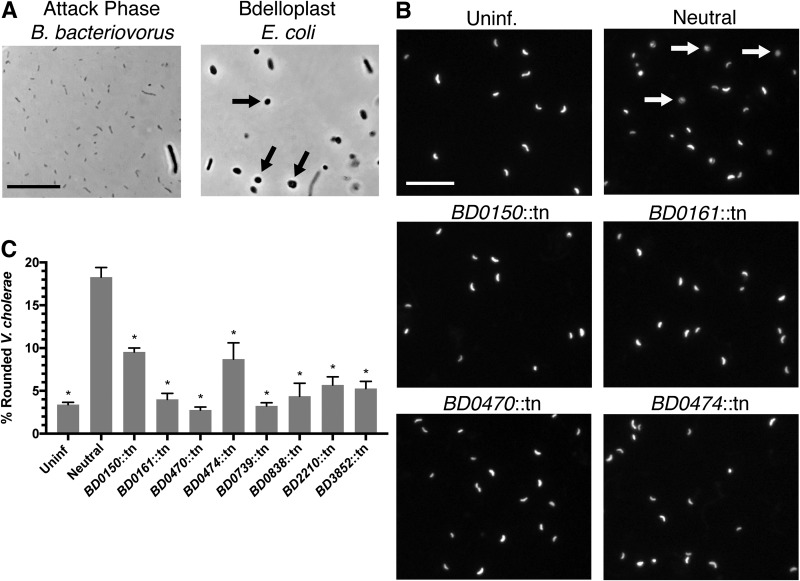
Identification of B. bacteriovorus mutants with defects in prey rounding by Tn-SphereSeq. (A) Microscopy images of attack-phase B. bacteriovorus and E. coli bdelloplasts isolated by differential centrifugation for Tn-SphereSeq. Arrows indicate bdelloplasts. (B) Fluorescence images of GFP-expressing V. cholerae 3 h following infection with B. bacteriovorus at an MOI of 1. Arrows indicate bdelloplasts. Scale bar = 10 μm. (C) The percentage of rounded V. cholerae cells was calculated by analyzing images by Matlab for roundness (eccentricity) of three biological replicates. Significance was determined by comparing each strain’s rounding percentage to that of the neutral control. *, *P < *0.0001 (ANOVA with Dunnett’s multiple-comparison test).

For this assay, we used the same B. bacteriovorus mutant library from Tn-FACSeq and E. coli WM3064 as the prey. We achieved better bdelloplast isolation with E. coli than with V. cholerae (Fig. 4A), and strain WM3064 is easily selected against, as it is a diaminopimelic acid (DAP) auxotroph (27). Following 3 h of infection, we resuspended the bacteria in a Percoll-sucrose solution and used ultracentrifugation to separate the B. bacteriovorus mutants by growth phase. We plated the inputs and prey bdelloplasts containing B. bacteriovorus mutants onto separate petri plates and allowed 8 days of growth before pooling the colonies. We next isolated gDNA and repeated the Tn-seq analysis as described above for six biological replicates (Data Set S5).

Rounding scores close to 1.0 indicate mutants that retain the ability to round their prey, and the intergenic control mutants collectively had a rounding score of 1.2. Of the 101 B. bacteriovorus mutants tested, 41 had rounding scores below 0.4, and 15 had rounding scores below 0.2. The majority of the top Tn-FACSeq hits tested also had low rounding scores (10/14), which was expected given that attachment is a prerequisite for prey rounding. Like the Tn-FACSeq hits, mutants with low rounding scores had insertions in pilus genes, adventurous gliding motility genes, and many hypotheticals.

To validate the Tn-SphereSeq results, we tested mutants of interest for prey rounding by microscopy. Previously, we developed an assay to test for prey rounding by WT B. bacteriovorus using GFP-expressing V. cholerae and a custom Matlab script that calculates the eccentricity (roundness) of individual cells (20). Axenic growth mutants of B. bacteriovorus are slower predators than the WT, so we observed prey rounding at 3 h postinfection. As expected, the neutral mutant rounded 18% of V. cholerae cells, while only 3% of uninfected V. cholerae cells scored as rounded (Fig. 4B and C). All B. bacteriovorus mutants tested showed significantly reduced prey rounding compared to the neutral control (*P = *0.0001). This included the *BD0474*::tn mutant, which appears to be able to attach to prey but unable to kill or round them (Fig. 2D, 3, and 4B and C). All other mutants showed defects in prey attachment, killing, and rounding, including several with insertions in hypothetical and uncharacterized genes. These data are summarized in Table 2.

**TABLE 2:** Classification and data summary for tested B. bacteriovorus mutants

Gene	Protein^*a*^	NCBI function	Activity by predation deficiency^*b*^			Class
			Attach	Kill	Round	
Bd0075	hypo	Unknown	**−**	**−**	ND	1
Bd0150	hypo	Unknown	**−**	**−**	**−**	1
Bd0161	hypo	Unknown	**−**	**−**	**−**	1
Bd0470	TadC	Type IVb pilus	**−**	**−**	**−**	1
Bd0474	hypo	Unknown	**+**	**−**	**−**	2
Bd0739	hypo	Unknown	**−**	**−**	**−**	1
Bd0838	AglS	Adventurous gliding motility	**−**	**−**	**−**	1
Bd2210	hypo	Unknown	**−**	**−**	**−**	1
Bd2490	hypo	Unknown	**−**	**−**	ND	1
Bd2684	hypo	Unknown	**−**	**−**	ND	1
Bd3852	PilT	Type IVa pilus	**−**	**−**	**−**	1

a hypo, hypothetical.

b **−**, no activity; +, activity; ND, not determined.

## DISCUSSION

For nearly 60 years, B. bacteriovorus has fascinated investigators due to its unusual predatory life cycle and potential as a therapy against Gram-negative bacterial pathogens. Until recently, however, B. bacteriovorus researchers lacked the tools for comprehensive forward-genetic screens, thus limiting our understanding of the gene requirements for predation. This changed in 2008 when two studies successfully used transposon mutagenesis to identify genes involved predator-prey interactions (17, 18). However, these studies screened fewer than 6,000 mutants in total, which is below saturation for the 3.8-Mb genome of B. bacteriovorus (29). To build on this work, we carried out a saturating Tn-seq screen and identified over 100 genes required for predation, including 66 hypothetical genes. We also created the first B. bacteriovorus ordered-knockout library and developed new high-throughput techniques to characterize mutants by stage of predation deficiency.

Of the 11 mutants selected for in-depth follow up, all except *BD0474*::tn showed significant reductions in predator-prey attachment and are class I mutants. The majority of these genes are hypothetical and uncharacterized, although three have assigned functions, *aglS* (*Bd0838*, adventurous gliding motility), *pilT* (*Bd3852*, type IVa pilus), and *tadC* (*Bd0470*, type IVb pilus). PilT was previously implicated in predation by Medina et al., and the type IVa pilus is hypothesized to play a role in predator attachment or invasion into the prey periplasm (17, 23). Adventurous gliding, or A-motility, is best described in Myxococcus xanthus yet has been shown to be important for B. bacteriovorus invasion of prey (30). In results somewhat contradicting ours, Avidan et al. recently demonstrated that certain type IVb pilus genes are required for predator invasion but dispensable for attachment (13). In our hands, the type IVb gene *tadC* (*Bd0470*) was required for attachment. However, it lies upstream in an operon with *Bd0474*, a class II mutant, which was not defective until after attachment and may be part of the type IVb pilus machinery.

Our Tn-seq data may yield additional information when paired with previous transcriptomics data. For example, Karunker et al. performed RNA-seq to identify B. bacteriovorus genes induced in attack or growth phase (31). When aligning these results to ours, we find 39% of genes from the mini-Tn-seq low-fitness hits (*w *<* *0.3) showed attack phase-induced gene expression, while 31% had increased expression during growth phase. These percentages were similar if the mutants were grouped by Tn-FACSeq or Tn-SphereSeq hits, suggesting that gene hits from the high-throughput follow-up assays do not necessarily correlate with growth stage-specific gene expression. These data, along with microarray expression data by Lambert et al., are included in Data Set S6 (32).

10.1128/mBio.01040-19.9 DATA SET S6 Tn-seq data supplemented with RNA-seq data from Karunker et al. (31) and microarray data from Lambert et al. (32). Download Data Set S6, XLS file, 0.1 MB. Copyright © 2019 Duncan et al. 2019 Duncan et al. This content is distributed under the terms of the Creative Commons Attribution 4.0 International license.

Other recent work provides genetic and phenotypic data to compare with our Tn-seq screen results. For example, several studies have explored the role of cyclic di-GMP (c-di-GMP) in B. bacteriovorus predation. Hobley et al. demonstrated that deleting the diguanylate cyclase gene *dgcB* (*Bd0742*) completely abrogated predation (33), and our *dgcB*::tn mutant strain was severely attenuated as well (*w *=* *0.0001). Similarly, this study found *dgcC* (*Bd1434*) to be essential for HI growth, and we did not observe any transposon insertions in this gene. In addition, Rotem et al. used mass spectroscopy to identify 84 putative c-di-GMP binding proteins in B. bacteriovorus. However, only six of these were confirmed to be required for predation by our mini-Tn-seq, *Bd0407*, *Bd0604*, *Bd0836*, *Bd1937*, *Bd2726*, and *Bd2872* (34).

One consideration for transposon screens is that genetic redundancy can prevent the identification of important pathways. For instance, previous studies have described *Bd0816*, *Bd3459*, *Bd0886*, and *Bd1176* as being required for B. bacteriovorus’ remodeling of prey peptidoglycan (16, 35). While these genes were not identified in our Tn-seq screens, Lerner et al. (35) and Kuru et al. (16) did not observe predator defects unless using double-knockout mutants. Furthermore, Dwidar et al. demonstrated five B. bacteriovorus proteases with high expression during intraperiplasmic growth (36). However, these genes were not found in our Tn-seq screens, likely due to genetic redundancy.

In addition to the many genes we found critical for predation, we also discovered several transposon mutants with improved fitness in our Tn-seq screens. One such mutant, *Bd0108*::tn, represents the Hit (host-interaction) locus known to regulate the type IVa pilus and HI growth (37). While it remains unclear if this predation advantage would translate to WT B. bacteriovorus or is an artifact of the HI strain background, follow-up studies should explore this possibility. Similarly, several genes validated as only important for killing certain prey species or prey states. These results could empower engineering of hyperefficient predators or customized B. bacteriovorus strains that distinguish between different pathogens or planktonic and biofilm prey.

In this study, we have broadly characterized over 100 B. bacteriovorus genes by stage of predation deficiency, and we propose a classification system, classes I to IV, to standardize the description of mutant phenotypes. Although we only identified mutants in classes I and II, we hypothesize that mutants in classes III and IV are possible and may be among the transposon mutants with reduced fitness that we did not characterize further. For instance, Hobley et al. found that deleting *dgcA* (*Bd0367*), which codes for a diguanylate cyclase, prevented exit from prey (33). Although more follow up on these genes is needed, the genome-wide fitness data we generated can serve as a resource and catalyst for future studies. We also anticipate that the ordered-knockout library of B. bacteriovorus will be a useful tool for us and others in the field.

## MATERIALS AND METHODS

### Tn-seq screens during B. bacteriovorus predation.

For the initial Tn-seq, B. bacteriovorus transposon mutants were pooled from petri plates. This pool was diluted and grown in 3 separate tubes of liquid PYE broth for 2 days in a roller drum at 30°C. The liquid-grown B. bacteriovorus pools were added to planktonic V. cholerae and E. coli cells at an MOI of 0.001 and added to biofilm prey at an MOI of 0.01. Planktonic prey were prepared by growing overnight at 30°C in a shaking incubator and resuspended the following day in 10 ml HEPES buffer. The infection mixtures were incubated in 125-ml flasks with shaking for 30 h (E. coli) or 48 h (V. cholerae) at 30°C, resulting in ∼99% of the prey being killed. Biofilm prey were prepared by diluting overnight prey cultures 1:100 into 15 ml of LB Miller broth in a petri plate and allowed to grow for 24 h (E. coli) or 48 h (V. cholerae) statically at 30°C (38). Following this growth, the biofilms were gently washed once with HEPES buffer, and 10 ml of HEPES buffer was added to each petri plate containing B. bacteriovorus transposon mutants. The infection was incubated statically for 30 h (E. coli) or 48 h (V. cholerae) at 30°C, resulting in ∼99% of the prey being killed. Detailed information on bacterial strains and growth conditions and on the generation and robotic arraying of the B. bacteriovorus transposon insertion mutant library is provided in Text S1.

10.1128/mBio.01040-19.3 TEXT S1 Description of strains, growth conditions, and B. bacteriovorus transposon insertion mutant array. Download Text S1, PDF file, 0.1 MB. Copyright © 2019 Duncan et al. 2019 Duncan et al. This content is distributed under the terms of the Creative Commons Attribution 4.0 International license.

Genomic DNA was isolated using the DNeasy blood and tissue kit (Qiagen catalog no. 69506), and the Tn-seq DNA was prepared for sequencing, as previously described (20). Primers for amplifying and indexing the samples are listed in Data Set S7.

10.1128/mBio.01040-19.10 DATA SET S7 Primers used in this study. Download Data Set S7, XLS file, 0.1 MB. Copyright © 2019 Duncan et al. 2019 Duncan et al. This content is distributed under the terms of the Creative Commons Attribution 4.0 International license.

### Sequencing and fitness calculation.

Pooled and indexed DNA samples were sequenced on the Illumina HiSeq 2500 platform using a Mariner-specific sequencing primer. The sequence reads of transposon junctions were analyzed using the Tufts University Core Facility (TUCF) Galaxy server, as previously described (20).

### Prey killing and competition assays.

Prey E. coli (MG1655) was grown overnight in LB Miller broth and diluted in HEPES buffer to an optical density at 600 nm (OD_600_) of 2.0, and 100 μl of prey bacteria was added to wells of a 96-well plate (Corning catalog no. 3788). Individual B. bacteriovorus mutants from the ordered-knockout library were added at an MOI of 1 for the prey killing assay and at an MOI of 0.001 for the complementation experiments. Complementation of the *BD0470*::tn and *BD0474*::tn mutants was incomplete via each gene’s native promoter and required 1 mM isopropyl β-d-1 thiogalactopyranoside (IPTG; VWR catalog no. 97061-778) to induce gene expression from the P_tac_ promoter present on the plasmid. Following 30 h of shaking at 30°C, we plated serial dilutions and counted surviving E. coli CFU the following day.

### Tn-FACSeq and flow cytometry.

B. bacteriovorus transposon mutants of interest, harboring pMMB207red, were grown overnight at 30°C in a roller drum to an OD_600_ of 0.2 to 0.4. Overnight cultures of GFP-expressing V. cholerae were resuspended in HEPES to 10^8^ CFU/ml. Fresh cultures of tdTomato-expressing B. bacteriovorus were washed twice with HEPES and added to the V. cholerae at an MOI of 1. At 3 h postinfection, the bacteria were processed using an S3e cell sorter (Bio-Rad).

For Tn-FACSeq, samples were sorted for 2 h into a red event-only tube (B. bacteriovorus) or a green and red-event tube (B. bacteriovorus attached to V. cholerae) using the S3e cell sorter (Bio-Rad). We used a maximum event rate of 1,000 events/s, since higher rates resulted in reduced viability of the sorted cells. The samples were then spread on PYE petri plates and grown for 8 days, and the resultant colonies were pooled for each biological replicate. We pooled at least 10,000 colonies per biological replicate for six biological replicates. Genomic DNA was isolated and the DNA prepared for sequencing as described above. The attachment score indicates the relative abundance of B. bacteriovorus mutants in the red-only pool compared to that in the green and red pool.

### Tn-SphereSeq.

We separated B. bacteriovorus growth phases as previously described (26). In brief, B. bacteriovorus transposon mutants of interest were grown overnight at 30°C in a roller drum to an OD_600_ of 0.2 to 0.4. Overnight cultures of DAP auxotroph E. coli WM3064 were resuspended in 40 ml of 25 mM HEPES buffer to 1.6 × 10^8^ CFU/ml and infected with B. bacteriovorus at 8 × 10^8^ CFU/ml. Following 3 h of infection at 30°C with shaking, the mixture was pelleted for 10 min at 20,000 × *g*. The pellet was next resuspended in a solution of five parts Percoll (Sigma-Aldrich catalog no. P1644) and four parts 0.25 M sucrose (Sigma-Aldrich catalog no. S7903) and centrifuged for 30 min at 50,000 × *g*. Next, the bdelloplasts were removed from a ring at the top of the tube and plated on PYE plates. The B. bacteriovorus inputs were simultaneously plated as well. Following 8 days of growth, we pooled 100,000 colonies from each of the six biological replicates and performed Tn-seq as described above. For the final analysis, we compared the inputs to bdelloplast population to identify B. bacteriovorus mutants defective in prey rounding relative to the bulk population.

### Fluorescence microscopy.

Overnight cultures of GFP-expressing V. cholerae were resuspended in HEPES to 10^8^ CFU/ml and infected with nonfluorescent B. bacteriovorus at an MOI of 1. Following 3 h of infection, the bacteria were fixed in 1% formaldehyde (Fisher Scientific catalog no. 28906) for 10 min. Slides were prepared and imaged as previously described (20). Between 2,500 and 7,500 bacteria were analyzed for each condition across the three biological replicates.

### Image analysis.

For the prey rounding experiment, images were analyzed as previously described (20). For the predator attachment experiment, images were scored in double-blind manner for invasion of B. bacteriovorus into V. cholerae or external attachment. For each mutant, we scored 1,100 B. bacteriovorus cells across three biological replicates.

### Statistical analysis.

All statistical analysis was done on GraphPad Prism by ordinary one-way analysis of variance (ANOVA) with Dunnett’s multiple-comparison test for significance.

### Data availability.

Information on the strains and primers used is included in Data Sets S1 and S6, respectively. Tn-seq results are included in Data Sets S2 to S5.

10.1128/mBio.01040-19.2 FIG S2 Flow cytometry controls and gating strategy for Tn-FACSeq validation experiments. The left panels gate all red events, and the right panels gate all events that are red and green from the same experiment. (A) GFP-positive V. cholerae. (B) TdTomato-positive B. bacteriovorus only. (C) Fluorescent V. cholerae and B. bacteriovorus mixed immediately before analysis. Download FIG S2, TIF file, 1.5 MB. Copyright © 2019 Duncan et al. 2019 Duncan et al. This content is distributed under the terms of the Creative Commons Attribution 4.0 International license.
