# Supplementary figures and images for: High-Throughput Analysis of Gene Function in the Bacterial Predator Bdellovibrio bacteriovorus
Source: mBio. 2019 Jun 11;10(3):e01040-19. doi: 10.1128/mBio.01040-19 (PMC6561027; doi:10.1128/mBio.01040-19)

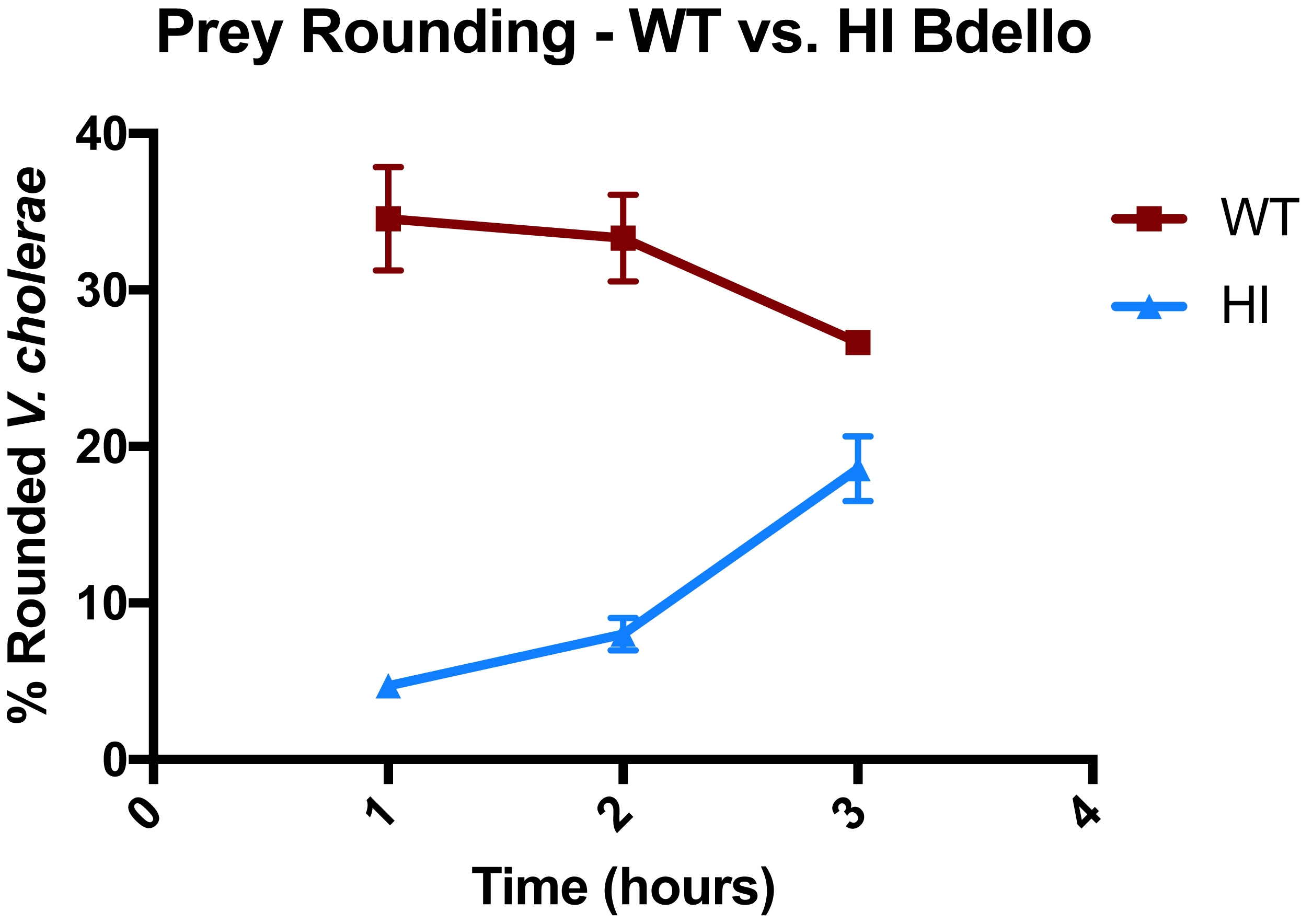

Supplement: FIG S1 [file mBio.01040-19-sf001.tif]

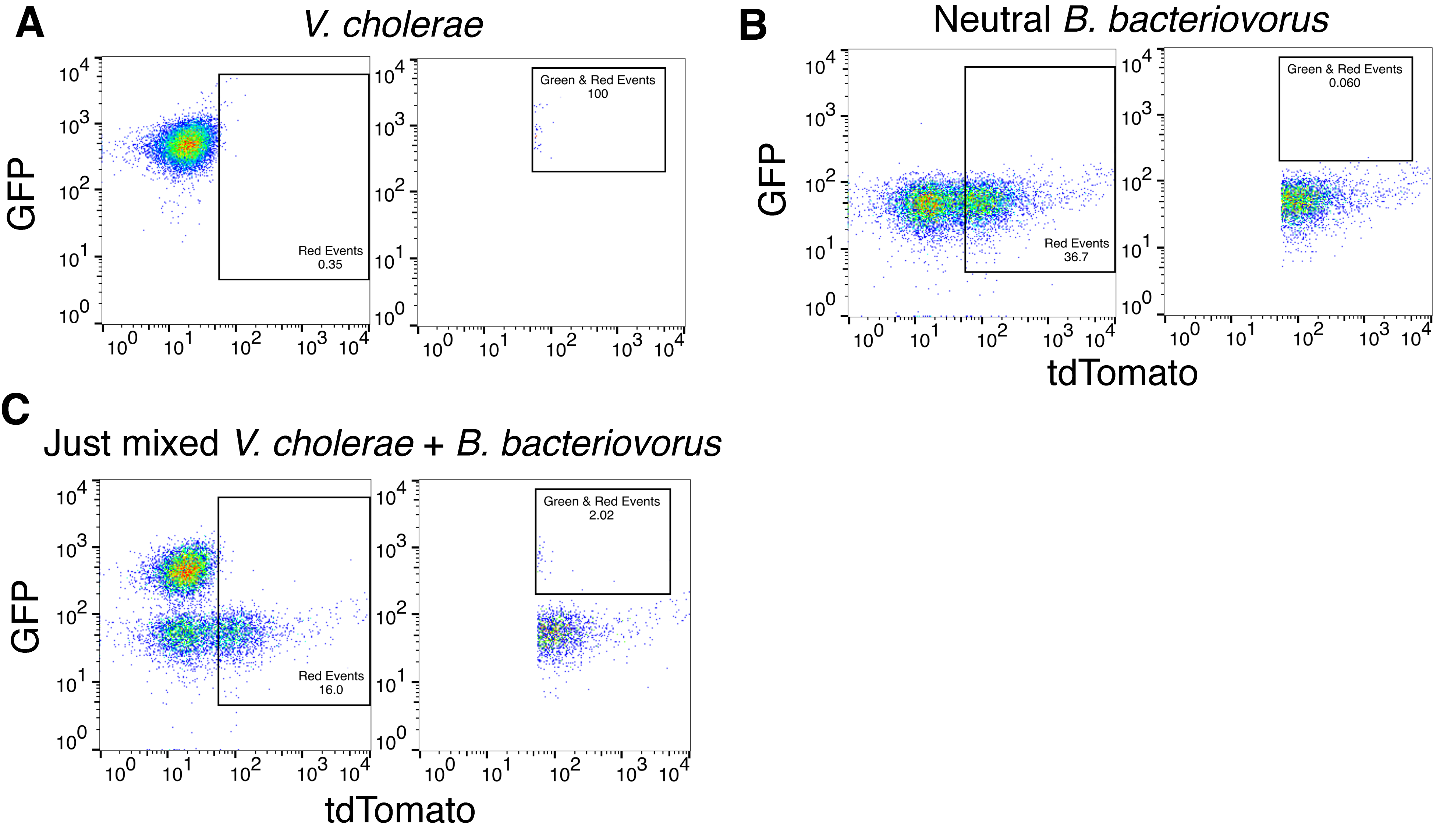

Supplement: FIG S2 [file mBio.01040-19-sf002.tif]
