# Supplementary material for: High-Throughput Analysis of Gene Function in the Bacterial Predator Bdellovibrio bacteriovorus
Source: mBio. 2019 Jun 11;10(3):e01040-19. doi: 10.1128/mBio.01040-19 (PMC6561027; doi:10.1128/mBio.01040-19)
Supplement: TEXT S1 [file mBio.01040-19-s0001.pdf]

Text S1. Supplemental Materials and Methods

Bacterial strains and growth conditions. Strains used in this study are listed in Data Set S1. A host-independent isolate of *B. bacteriovorus* 109J was used as the parent strain for all experiments (38). *B. bacteriovorus* strains were grown on Peptone Yeast Extract (PYE, BD 211677, Sigma-Aldrich Y1625) plates, supplemented with 3mM magnesium chloride (Sigma-Aldrich M2670) and 2mM calcium chloride (Sigma-Aldrich C5080). Following 4-6 days of growth on plates at 30°C, strains were grown in liquid PYE supplemented with 3mM magnesium chloride and 2mM calcium chloride overnight at 30°C in a rollerdrum or shaking incubator. Gentamicin (Sigma-Aldrich G1914) was used at 10 μg/ml for *B. bacteriovorus* transposon mutants. Chloramphenicol (Fisher Scientific BP904) at 5 μg/ml was used for *B. bacteriovorus* carrying the fluorescent plasmid pMMB207red or strains complemented with pMMB207. Complemented strains were created by cloning genes of interest and their native promoters into pMMB207. A *V. cholerae* O1 serogroup, Ogawa serotype, El Tor biotype strain isolated from Haiti in 2015 and *E. coli* MG1655 were used as prey in this study (20). *V. cholerae* and *E. coli* were grown overnight at 30°C with shaking in LB Miller broth (Fisher Scientific BP1426). All predation experiments were carried out in 25 mM HEPES buffer (Fisher Scientific 11344-041) supplemented with 3 mM magnesium chloride and 2 mM calcium chloride.

Generation and arraying of *B. bacteriovorus* transposon insertion mutant library. *B. bacteriovorus* transposon mutants were created as previously described (17). Following 7 days of growth, the *B. bacteriovorus* colonies were scraped up used immediately for use in Tn-seq or frozen in 20% glycerol for later use. The final library consisted of over 90,000 unique transposon mutants.

To create the arrayed knock-out library, aliquots of the frozen transposon library were spread onto large Q-Tray petri plates (Molecular Devices SKU# X6023), and grown for seven days. Individual colonies were picked using a Genetix QPExpression automated colony and array picker and inoculated into 384-well plates (Corning 3680) containing PYE broth with 10 μg/ml gentamicin. The resulting arrayed library consisted of 68 unique plates and was incubated at 30°C for 48 hours. Each plate was replicated to yield a backup library. Following growth, glycerol was added to a final concentration of 20%, and the plates were stored at -80°C.

Using previously published techniques (24, 25), insertion mutants of interest were located in the array using a combinatorial pooling method that enabled insertion sites to be correlated with clone location. To identify the location of individual transposon insertions, the arrayed library was thawed, and a Tecan Freedom Evo 200 liquid handling robot system was used to generate 24 predetermined combinatorial libraries from 13,056 strains picked from the first 34 plates, wherein each strain was added to a unique combination of the 24 libraries. Genomic DNA (gDNA) was then isolated from these 24 pools, processed, barcoded, and deep sequenced as described below for Tn-seq experiments. This process was repeated for the second set of 34 plates, and the resulting reads from each pool were mapped to the *B. bacteriovorus* reference genome CP007656.1 using Bowtie2. The resulting SAM files for each of the two sets of 24 pools of combinatorial libraries were used as an input for a custom Perl script that correlates insertion sites with individual clones in specific wells based on the pools in which the mutants were present. Specific insertions were preliminarily assigned to about 35% (9,000) of the wells. The remaining 65% of wells could not be accurately assigned due to technical problems encountered during the inoculation of clones into individual wells. Approximately 90% of the assigned mutants were confirmed by colony PCR.
